# Anti-acne potential of *Haematococcus pluvialis*: GC–MS profiling and antibacterial activity of n–hexadecanoic acid and α–tocopherol

**DOI:** 10.5455/javar.2025.l958

**Published:** 2025-09-22

**Authors:** Siti Hudaidah, Muhammad Kholiqul Amiin, Diki Danar Tri Winanti, Maulid Wahid Yusup, Arief Rahman Rivaie, Syifania Hanifah Samara, Linda Ratna Sari, Gregorius Nugroho Susanto

**Affiliations:** 1 Department of Aquaculture, Faculty of Agriculture, Universitas Lampung, Bandar Lampung City, Indonesia; 2 Department of Marine, Faculty of Agriculture, Universitas Lampung, Bandar Lampung City, Indonesia; 3 Department of Agricultural Product Technology, Faculty of Agriculture, Universitas Lampung, Bandar Lampung City, Indonesia; 4 Research Center for Fishery, National Research and Innovation Agency (BRIN), Cibinong, Indonesia; 5 Department of Aquaculture, Faculty of Fisheries and Marine, Universitas Airlangga, Surabaya, Indonesia; 6 Department of Biology, Faculty of Mathematics and Natural Sciences, Universitas Lampung, Bandar Lampung City, Indonesia

**Keywords:** Microalgae, anti–acne, bioactive compound, Haematococcus pluvialis, GC-MS profiling, n-hexadecanoic acid

## Abstract

**Objective:**

This study explores the bioactive compounds in *Haematococcus pluvialis* and their potential as anti-acne agents through gas chromatography–mass spectrometry (GC–MS) analysis and disc diffusion assays.

**Materials and Methods:**

3.3 gm of *H*. *pluvialis* was extracted and tested against three acne-causing pathogens: *Propionibacterium acnes*, *Staphylococcus aureus*, and *Staphylococcus epidermidis*, under varying extract concentrations.

**Results:**

The GC–MS analysis detected several bioactive compounds in the extract, including fatty acids (e.g., n-hexadecanoic acid and linoleic acid esters), polysaccharides, vitamins, aromatic compounds, and diterpenoids. Fatty acids and vitamin compounds dominated, comprising 72.20% of the extract, with alpha-tocopherol and its derivatives as prominent components. Antimicrobial activity was assessed using the disc-diffusion method. The extract demonstrated notable antibacterial effects, particularly at 10 ppm, achieving inhibition zones of 18.0 ± 2.0 mm against *P*. *acnes*, 19.9 ± 1.7 mm against *S. aureus*, and 19.5 ± 4.7 mm against *S*. *epidermidis*. These zones of inhibition were classified as intermediate.

**Conclusion:**

The results indicate that microalgae from the Chlorophyceae class are more effective against Gram-positive than Gram-negative bacteria. These findings emphasize the potential of bioactive compounds as anti-acne agents, suggesting they could serve as substitutes for antibiotics.

## Introduction

Acne is a long-term inflammatory condition of the hair follicle and sebaceous glands (oil-producing glands) [1]. Comedones, papules, pustules, nodules, or cysts clinically characterize it. Acne can also cause complications such as scarring and changes in skin pigmentation. Additionally, it negatively affects quality of life, leading to anxiety, depression, and a loss of self-esteem [2]. The disorder impacts over 80%–85% of adolescents and young adults. However, it can persist into adulthood, especially for women. More than 40% of women experience acne after age 25 [3].

Epidemiologically, in Indonesia, acne is the most common skin disorder; characteristics can vary based on factors such as demographics, race/ethnicity, and regional differences in the level of pathogenicity [4]. According to the Indonesian Cosmetic Dermatology Study, females aged 14–17 years have a prevalence of *Acne vulgaris* of 83%–85%, with rates of 95%–100% in males aged 16–19 years [5].

Research conducted in Lampung shows that acne occurs more frequently in women (69.7%) than in men (30.3%). The younger age group (16–25 years old) has a higher risk of acne, at 53.2%. Those using cosmetics were more likely to experience acne (59.1%). Although the prevalence in Lampung is quite high, an epidemiological overview shows that this disease is more common in young women (16–25 years) [6].

A major cause of acne can be bacterial infection. *Propionibacterium acnes*, *Staphylococcus aureus*, and *Malassezia furfur* are involved in the development of acne. Although *M. furfur* is recognized as a factor, it was excluded from this study because of its fungal nature, which requires different culture media and specialized growth conditions, unlike those needed for bacterial pathogens. This research focused on bacterial acne pathogens, mainly *S. aureus* and *P. acnes*, which are more commonly used in antibacterial screening tests. *S. aureus *is part of the normal skin flora, especially on the face, and is classified as a Gram-positive bacterium. However, it is not part of the Corynebacterium group, as these are distinct genera within Gram-positive bacteria [7]. Using antimicrobial or anti-acne agents is an effective way to manage acne linked to bacterial infection. Commercial anti-acne products are usually based on antibiotics. The use of antibiotics for acne treatment has raised concerns, especially regarding antimicrobial resistance. Additionally, antibiotics may cause side effects like redness, irritation, itching, and swelling [7].

In terms of overcoming acne problems, nature offers potential solutions in skincare products. Indonesia, renowned for its rich biodiversity, is home to a wide variety of marine organisms, including *H. pluvialis*. This microalgae is valued for its antioxidant properties and is used for acne treatment [8]. *Haematococcus* *pluvialis *is a single-celled green microalgae in freshwater, classified in the Chlorophyceae class. It has attracted considerable interest from both the scientific and biotechnology communities as one of the most important biological sources of the carotenoid astaxanthin in nature [9,10]. Under extreme environmental conditions, such as high-intensity light or oligotrophic circumstances, these species undergo morphological and biochemical modifications, including intense carotenoid biosynthesis, and reach up to 4.0% of cell dry weight [11]. Therefore, considering the bioactive potential of *H*.* pluvialis* as an antimicrobial and anti-acne agent, its application as an antibacterial treatment for acne highlights the feasibility of encapsulating its carotenoid-rich extract into poly lactic–co–glycolic acid nanocapsules. Carotenoids in the nanocapsule formulation showed higher antioxidant potential when compared to ascorbic acid [8].

In Indonesia,* H*. *pluvialis *has been extensively studied as a raw material for anti-acne treatments, serving as an alternative to antibiotics for treating bacterial infections in humans, animals, and fish. One of the compounds in *H*. *pluvialis* is astaxanthin. Astaxanthin in algae contains 10,000–50,000 ppm. Astaxanthin is a feed additive, cosmetic ingredient, pharmaceutical, and medicinal material. In addition to astaxanthin, *H*. *pluvialis* accumulates other valuable compounds with commercial potential, such as carbohydrates, lipids, and proteins. This study aims to evaluate the potential and resistance of microalgae extracts *in vitro* as an antiacne agent.

## Materials and Methods

### Ethical approval

This study did not require ethical approval; however, all samples were collected by standard sample collection procedures following the research ethics.

### Culture of microalgae

**Figure 1.**
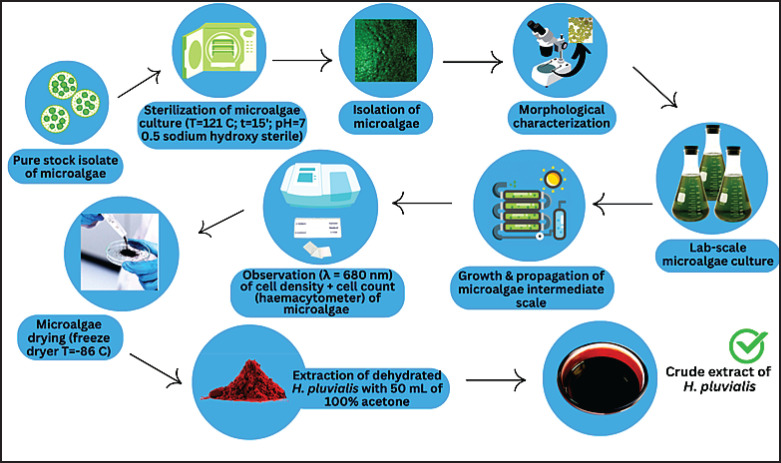
Microalgae culture and extraction process.

The materials used in this study include a culture of *H. pluvialis* microalgae (sourced from Kendal, Central Java), Walne’s medium without vitamin B12, sterile 0.5 M sodium hydroxide (NaOH), and 0.8% (w/v) sodium chloride (NaCl) solution. Figure 1 shows the flowchart of *H. pluvialis* culture and extraction.

The culture process begins with the preparation of equipment and materials, including a photobioreactor, hemocytometer, microcentrifuge, micropipette, microtubes, cover glass, microscope slides, F/2 Guillard medium, and Walne medium, with the potential of Hydrogen (pH) adjusted to a range of 7.5–8.0. These media are sterilized using an autoclave. Subsequently, *H*. *pluvialis* starter cultures were inoculated into the sterile medium at 10% of the total culture volume under aseptic conditions. Morphological examination at the microscope is necessary for characterization. Furthermore, laboratory-scale culture of the microalgae was conducted. In the vegetative phase, cultures are maintained under light intensities of 50–150 µmol/m^2^/sec with either a 12–h light/12–h dark cycle or a 16–h light/8–h dark cycle, at an optimal temperature of 20°C–25°C. Aeration was provided at a flow rate of 0.5–1 l/min, or cultures were agitated using a shaker at 100–150 rpm. The pH of the medium was continuously monitored and maintained within the range of 7.0–8.0. These conditions are also applied for microalgae culture on an intermediate scale using a photobioreactor. Stress conditions were applied to induce astaxanthin production by increasing the light intensity to 500–700 µmol/m^2^/sec, utilizing nitrogen-depleted media, adding NaCl to achieve a concentration of 0.5%–1% (w/v), and raising the temperature to 28°C–30°C.

Cultures were maintained until the vegetative phase reached 7–10 days or until optimal astaxanthin production at 10–15 days. Next, the measurement was done using a spectrophotometer and a hemocytometer. The samples were then stored in a freezer for drying for extraction, and collection. The extraction process of dried *H*. *pluvialis* was done by adding 50 ml of 100% acetone. After the extraction, the microalgae can be collected by centrifugation at 3,000–5,000 rpm for 5–10 min or by filtration. All steps are performed by maintaining sterile conditions to avoid contamination and ensure culture success.

### Analysis of gas chromatography–mass spectrometry (GC–MS)

Analysis was carried out using a capillary column model number: Agilent 19091S–433 HP–5MS, 5% Phenyl Methyl Siloxane (30 m × 0.25 mm, film thickness 0.25 µm). Helium served as the carrier gas, flowing at 1 ml/min, with the injector temperature set to 325°C. The injection mode was split, with a temperature of 290°C, a pressure of 7.58 psi, a split ratio of 50:1, and a total flow rate of 53.6 ml/min. Compound splitting in the GC column was managed using a temperature program that started at 50°C (held for 2 min), then increased by 10°C per minute until reaching 290°C, where it was held for 20 min. Mass spectrometry (MS) analysis was performed in electron ionization mode at an ionization energy of 35 eV. The ionization source temperature was maintained at 230°C, and the mass spectrum was acquired over a range of 50 to 550 m/z. An injection of up to 1 µl of the sample was made into the GC–MS system. The compounds in the extract were separated based on volatility in the GC column and identified via the MS detector. Identification was performed by matching retention times and detected compounds to standard libraries such as the National Center for Biotechnology Information (NCBI). Results were processed using GC–MS software to generate chromatograms. Target compounds were further analyzed based on retention time, fragmentation pattern, and peak intensity.

### Media preparation and bacterial suspension

Mueller–Hinton agar medium (Oxoid^®^ CM0337) was prepared by adding 38 gm of media powder per liter of distilled water in an Erlenmeyer tube (Pyrex^®^ 15 ml) and then heating until homogenized. The media were sterilized using an autoclave (Hirayama HVE–50) at 121°C for 15 min. The sterilized media was poured into Petri dishes (Pyrex^®^ 90 mm diameter, sterile) until it reached a thickness of 4 mm, and then it set.

Colonies of test bacteria were collected from pure cultures with the test bacteria used, i.e., *P. acnes* The American Type Culture Collection (ATCC)^®^ 11827, *Staphylococcus epidermidis* The Food and Nutrition Culture Collection^®^ 0048, and *S. aureus* ATCC^®^ 25923, then suspended in physiological NaCl solution (0.85%) and adjusted for turbidity with McFarland standard 0.5, equivalent to 1.5 × 10^8^ CFU/ml.

### Test disc preparation

**Figure 2.**
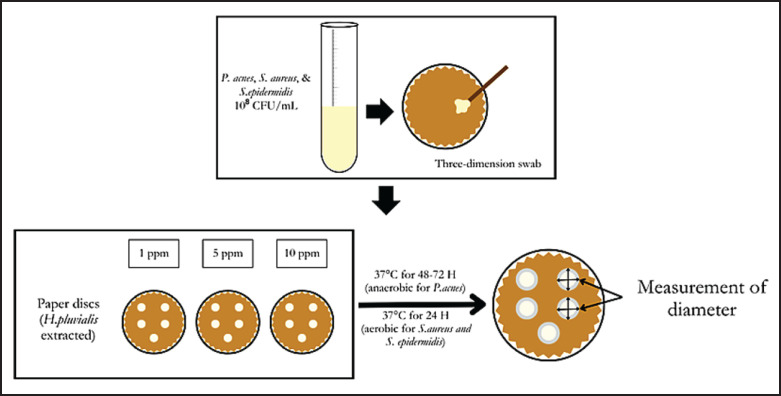
A disc diffusion assay of *H. pluvialis* extracted on *P. acnes*, *S. aureus*, and *S. epidermidis*.

The sterile paper discs (Oxoid^®^ Antimicrobial Susceptibility Discs (6 mm diameter) were soaked in *H. pluvialis* extract solutions with concentrations of 1, 5, 10 ppm, positive control (chloramphenicol 1,000 ppm), and solvent control (methanol) for 30 min. After being submerged, the discs were dried at room temperature in a sterile container until ready for use (Fig. 2).

### Disc diffusion assay procedure

The surface of the hardened Mueller–Hinton agar medium (Oxoid^®^ CM0337) is inoculated with the test bacterial suspension using a sterile cotton swab dipped in the bacterial suspension. The cotton swab was evenly wiped across the agar surface in a zigzag pattern to ensure homogeneous distribution. The prepared assay discs were placed on the surface of the media using sterile tweezers (Aesculap^®^), with a minimum distance between discs of 20 mm to prevent overlapping of inhibition zones. Positive and negative controls were also placed on the media for each test bacterium. The inoculated Petri dishes (Pyrex^®^ 90 mm diameter, sterile) were incubated in the incubator (Memmert^®^ IN55) under the following conditions: *P. acnes*: incubate at 37°C under anaerobic conditions for 48–72 h. *Staphylococcus epidermidis* and *S. aureus*: Incubate at 37°C under aerobic conditions for 24 h.

### Data analysis

After the incubation period, we measured the size of the inhibition zone (the clear area surrounding the disc) using a caliper with a precision of 0.1 mm. Results were measured by millimeters (mm) and logged. Each treatment was conducted three times to confirm the validity of the data. The mean diameter of the inhibition zone was calculated and then statistically analyzed using one-way ANOVA and post-hoc tests (Tukey HSD) to determine significant differences between treatment groups.

## Results

The results of the present study indicate that *H*. *pluvialis* contains several bioactive compounds with potential applications in biotechnology, pharmaceuticals, and medicine. The results in this study will report on the characterization of bioactive compound test results through GC–MS and disc diffusion assay using bacterial isolate samples of *P. acnes* ATCC^®^ 6919, *S. epidermidis* ATCC^®^ 12228, and *S. aureus* ATCC^®^ 25923.

### GC–MS assay

The samples of microalgae were analyzed. Data on bioactive compounds, content values, retention times, and molecular weights are presented in Table 1. Seventeen bioactive compounds were identified from the *H. pluvialis* sample during the analysis. Overall, two compounds were found to have the largest content of 17 compounds, namely n-hexadecanoic acid and α-tocopherol (Table 1). Based on the results, the identified bioactive compounds are classified into seven groups: fatty acids, polysaccharides, vitamins, aromatic compounds, diterpenoids, and other miscellaneous compounds.

Bioactive compounds found in *H*.* pluvialis* that include fatty acids are n–hexadecanoic acid (39.69%); 9, 12, 15–octadecatrienoic acid, methyl ester (1.982%); Linoleic acid ethyl ester (1.05%); 9, 12, 15–octadecatrienoic acid, ethyl ester (1.77%); 9(E), 11(E)–conjugated linoleic acid, ethyl ester (0.98%); and (2,7–octadienyl) succinic anhydride (0.68%). Besides fatty acids, polysaccharides are also found to be bioactive compounds. Those belonging to the polysaccharides group in the *H*. *pluvialis* extract sample were N–(2–2-methoxyphenyl) thiophene–2–sulfonamide (0.41%). The study found that the bioactive compounds other than fatty acids and polysaccharides in our sample were vitamins. The *H*. *pluvialis* extract sample contained two vitamins: alpha-tocopherol and alpha-tocopherol acetate (15.27% and 10.27%, respectively).

The bioactive compounds found in *H*.* pluvialis* that include aromatic compounds are 1,4–Bis(trimethylsilyl) benzene (1.04%); Indole–2–one, 2,3–dihydro–N–hydroxy–4–methoxy–3,3–dimethyl (2.12%); and 2–(Acetoxymethyl)–3–(methoxycarbonyl)biphenylene (2.09%). The other bioactive compound in *H*. *pluvialis* extract in the diterpenoid group is phytol (1.75%). The bioactive compound in *H*. *pluvialis*, including DEPH, is Bis (2–ethylhexyl) phthalate (2.63%). This study also found other bioactive compounds such as Cycloheptasiloxane, tetradecamethyl (1.48%); 9,12,15–octadecatrien–1–ol (8.94%); and Chondrillasterol (7.29%).

### Disc diffusion assay

**Figure 3.**
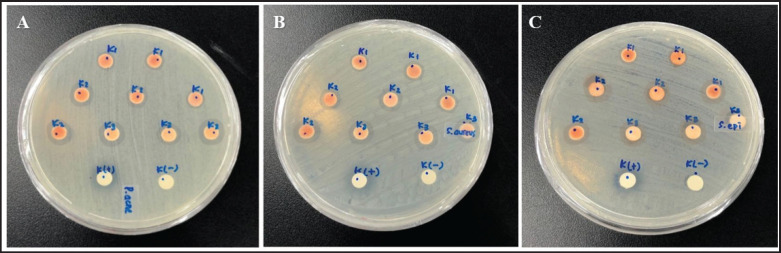
Results of the zone of inhibition for the antimicrobial assay. Description. (A): *Propionibacterium acnes*; (B): *Staphylococcus aureus*; (C): *Staphylococcus epidermidis*; K1: Concentration 1 ppm; K2: Concentration 5 ppm; K3: Concentration 10 ppm; K+: Positive control with chloramphenicol 1,000 ppm; K–: Negative control with methanol.

A disc diffusion assay is a commonly used method to evaluate the antibacterial activity of *H. pluvialis* extracts. This study aimed to investigate the antibacterial properties of paper discs against *P*. *acnes*, *S. aureus*, and *S*. *epidermidis*. The results of the disc-diffusion assay are presented in Figure 3 and Table 2.

In addition, to determine the different dose effects of each treatment on the inhibition zone of acne-causing bacteria (*P*. *acnes*, *S. aureus,* and *S*. *epidermidis*), data from the study were tested with analysis of variance (ANOVA). The ANOVA results are shown in Table 3.

The results of the ANOVA test indicated that the Fcal was 112.178, meaning that Fcal is greater than alpha 5%, or 0.05, with an Ftab of 3.48 (Fcal > Ftab 5%). It indicated a significant difference among the treatments in the provision of different doses of *H. pluvialis* extracts on the inhibition zone of *P*. *acnes*. Besides, the results of the ANOVA test for *S. aureus* and *S*.* epidermidis* showed that the Fcal was 61.961 and 29.605, meaning that Fcal is greater than alpha 5%, or 0.05, with an Ftab of 3.48 (Fcal > Ftab 5%). It indicated a significant difference among the treatments in the provision of different doses of *H. pluvialis* extracts on the inhibition zone of *S. aureus* and *S*.* epidermidis*.

Based on these results, it is necessary to analyze by post-hoc tests using the Tukey HSD test to determine the differences in each treatment. Tukey HSD test results are shown in Tables 4–6.

The post-hoc analysis by Tukey HSD indicated that the 1,000 ppm positive control (chloramphenicol) treatment had significantly greater antibacterial activity compared to all other concentrations and the methanol solvent control (*p* < 0.05). The largest difference was observed between 1,000 ppm and the solvent control (mean difference = 23.333 mm), indicating that the chloramphenicol exhibited strong antibacterial potential at higher concentrations. There were also significant differences between lower concentrations: 1 versus 5 ppm (mean difference = –5.166 mm, *p* = 0.0101) and 1 versus 10 ppm (mean difference = –8.533 mm, *p* = 0.0011). The most effective concentration among 1, 5, and 10 ppm was 10 ppm. The Tukey HSD test revealed that 10 ppm produced significantly greater inhibition compared to 1 ppm (*p* = 0.0011), indicating a statistically supported antibacterial effect. Although 10 ppm did not differ considerably from 5 ppm (*p* = 0.101), it showed a numerically higher inhibition zone, suggesting greater biological activity. These findings demonstrate a clear dose-dependent response, where higher concentrations of *H*. *pluvialis* extract produced larger zones of inhibition against *P*. *acnes*.

Among the Tukey HSD tested concentrations of *H*. *pluvialis* extract, 10 ppm did not differ significantly from 1,000 ppm statistically (*p* = 0.119); it still produced a large inhibition zone, suggesting that it retains considerable biological activity. This observation points to the possibility that, beyond a certain concentration, increases in extract dosage may not proportionally enhance the antibacterial effect. When examining the lower concentrations, 10 ppm emerged as the most promising submaximal dose. It significantly outperformed 1 ppm (*p* = 0.0011), and although the difference with 5 ppm was not statistically significant (*p* = 0.284), its larger inhibition zone indicates a better antibacterial response overall. In contrast, 1 ppm consistently produced the weakest response, affirming its limited efficacy at such a low concentration.

**Table 1.** GC–MS analysis of bioactive compounds in *H. pluvialis *extract.

Serial No	Bioactive compounds	Area %	Retention time (min)	Molecular weight (gm/mol)	Molecular structure	Chemical structure	Classes of bioactive compounds identified
1	N–(2–Methoxyphenyl) thiophene–2–sulfonamide	0.419%	9.409	335.4	C_14_H_13_N_3_O_3_S_2_	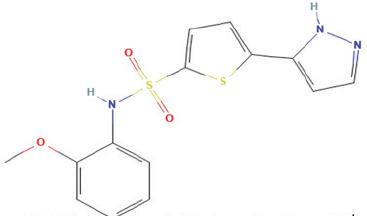	Polysaccharide
2	Cycloheptasiloxane, tetradecamethyl	1.484%	11.769	519.0	C_14_H_42_O_7_Si_7_	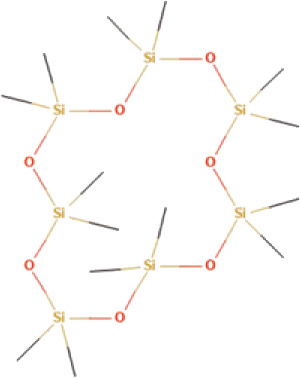	Other bioactive
3	n–Hexadecanoic acid	39.690%	17.531	600	C_16_H_32_O_2_	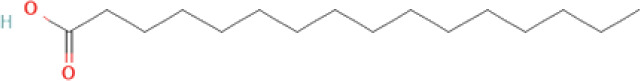	Fatty acid
4	9,12,15–Octadecatrienoic acid, methyl ester	1.982%	18.624	292.5	C_19_H_32_O_2_	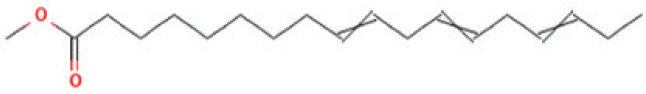	Fatty acid
5	Phytol	1.753%	18.747	271	C_20_H_40_O	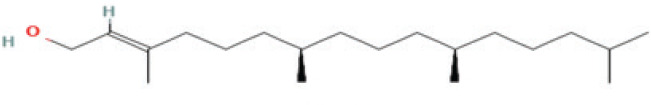	Diterpenoid
6	Linoleic acid ethyl ester	1.058%	19.198	308.5	C_20_H_36_O_2_	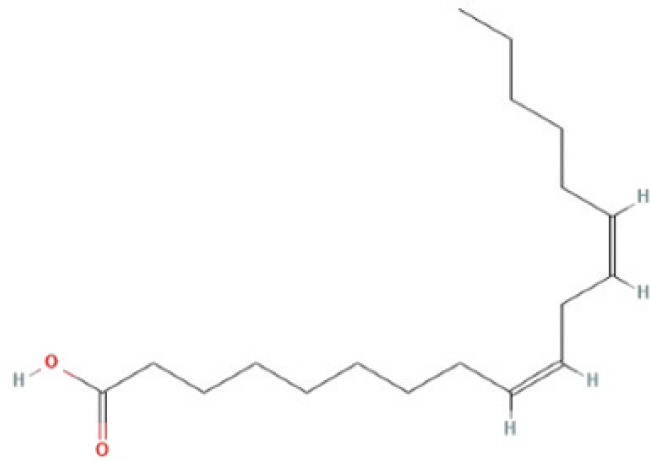	Fatty acid
7	9,12,15–Octadecatrienoic acid, ethyl ester	1.776%	19.268	306.5	C_20_H_34_O_2_	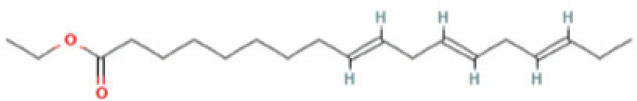	Fatty acid
8	9,12,15–Octadecatrien–1–ol	8.941%	20.030	264.4	C_18_H_32_O	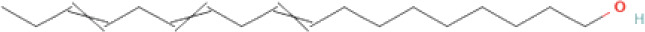	Other bioactive
9	9(E),11(E)–Conjugated linoleic acid, ethyl ester	0.987%	20.243	294.5	C_18_H_34_O_2_	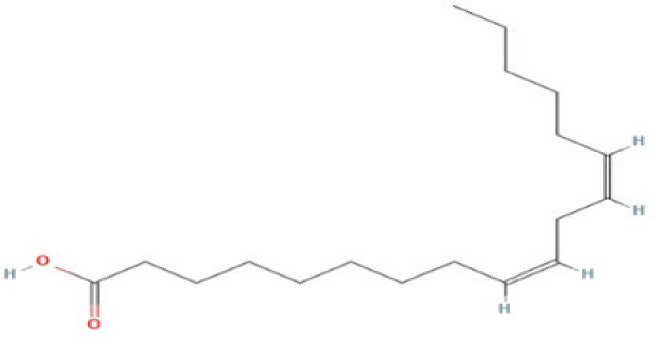	Fatty acid
10	(2,7–Octadienyl) succinic anhydride	0.683%	20.693	208.2	C_12_H_16_O_3_	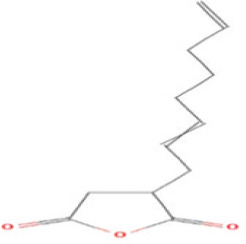	Fatty acid
11	Bis(2–ethylhexyl) phthalate	2.638%	22.619	243.4	C_24_H_38_O_4_	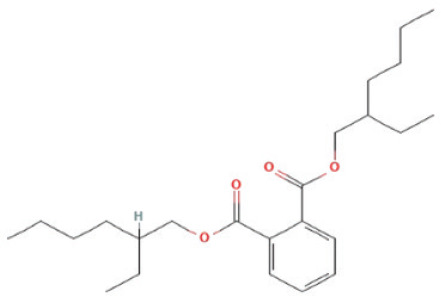	DEPH
12	1,4–Bis(trimethylsilyl)benzene	1.043%	24.813	222.4	C_12_H_22_Si_2_	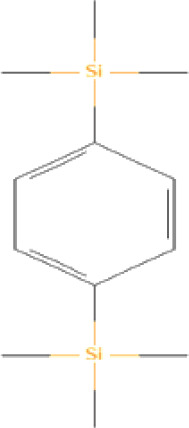	Aromatic compound
13	Alpha Tocopherol	15.755%	28.333	430.7	C_29_H_50_O_2_	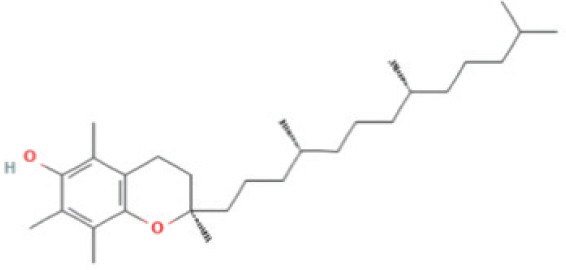	Vitamin
14	Alpha Tocopherol acetate	10.273%	29.190	472.7	C_31_H_52_O_3_	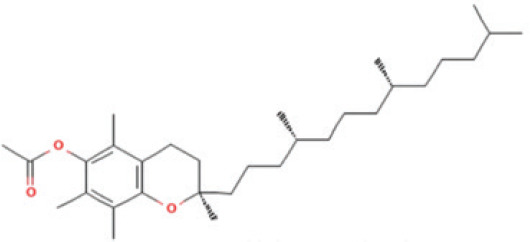	Vitamin
15	Indole–2–one, 2,3–dihydro–N–hydroxy–4–methoxy–3,3–dimethyl	2.126%	29.796	207.2	C_11_H_13_NO_3_	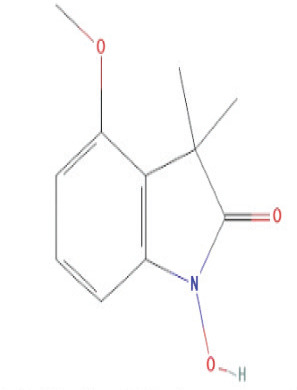	Aromatic compound
16	2–(Acetoxymethyl)–3–(methoxycarbonyl)biphenylene	2.092%	30.882	282.2	C_17_H_14_O_4_	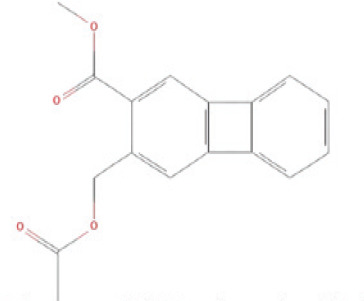	Aromatic compound
17	Chondrillasterol	7.297%	31.542	412	C_29_H_48_O	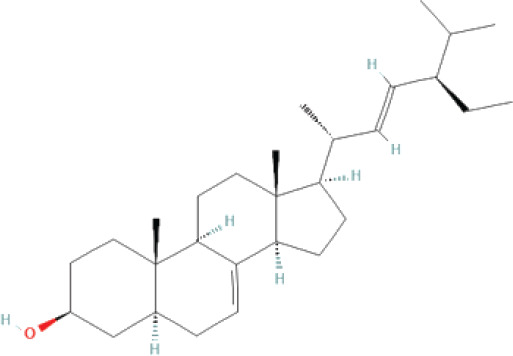	Other bioactive

**Table 2.** Antibacterial activity of *H. pluvialis* extract against *P. acnes, S. aureus*, and *S. epidermidis.*

Bacteria	Positive control(Chloramphenicol 1000 ppm)	Negative control (Methanol)	Concentration(ppm)	The average diameters of the inhibition zones (mm) (mean SD)
*P. acnes*	23.27 ± 1.5	0	1	9.47 ± 1.5
			5	14.6 ± 1.4
			10	18.0 ± 2.0
*S. aureus*	24.67 ± 2.3	0	1	8.63 ± 1.5
			5	16.2 ± 3.5
			10	19.9 ± 1.7
*S. epidermidis*	23.31 ± 1.5	0	1	10,0 ± 3.0
			5	15.2 ± 2.7
			10	19.5 ± 4.7

Note: The volume of the sample, positive control, and solvent control is 10 µl

**Table 3.** Results of the ANOVA test for the effect of different doses on the inhibition zone of *P. acnes, S. aureus*, and *S. epidermidis.*

Species	Source of variance	Sum of squares	*df*	Mean square	F_cal_	F_0.05_
*P. acnes*	Beetween Groups	947.677	4	236.919	112.178^1)^	3.48
	Within Groups	21.120	10	2.112	NA	NA
	Total	968.797	14	NA	NA	NA
*S. aureus*	Beetween Groups	1134.471	4	283.618	61.961^1)^	3.48
	Within Groups	45.773	10	4.577	NA	NA
	Total	1180.244	14	NA	NA	NA
*S. epidermidis*	Beetween Groups	990.083	4	247.521	29.605^1)^	3.48
	Within Groups	83.607	10	8.361	NA	NA
	Total	1073.689	14	NA	NA	NA

Note: ^1)^ Fcal > Ftab 0.05 indicates that it is significantly different (*p* < 0.05). NA, not applicable.

Table 6 showed that the antibacterial potential of *H*. *pluvialis* extract against *S. epidermidis* was assessed across various concentrations, excluding the positive control. 10 ppm appeared more promising than 1 ppm, with a statistically significant difference observed between them (*p* = 0.0161). This implies that increasing the dose from 1 ppm to 10 ppm substantially improves antibacterial activity. Overall, the findings suggest that 10 ppm offers an effective balance between activity and dosage, making it a viable candidate for further formulation studies, especially in applications where minimal concentration is preferred for cost or formulation efficiency for therapeutic or cosmetic applications targeting *P*. *acnes*, *S. aureus*, and *S*. *epidermidis*.

**Table 4.** Results of the tukey honestly significant differences (HSD) test for different doses on the inhibition zone of *P. acnes.*

Species	Treatment	Mean difference	Sig.	Indicators	Significant
*P. acnes*	1,000 ppm × metanol	23.333	0.001^1)^	Sig < 0.05	Yes
	1,000 × 1 ppm	13.866	0.001^1)^	Sig < 0.05	Yes
	1,000 × 5 ppm	8.700	0.001^1)^	Sig < 0.05	Yes
	1,000 × 10 ppm	5.333	0.008^1)^	Sig < 0.05	Yes
	1 × 5 ppm	–5.166	0.010^1)^	Sig < 0.05	Yes
	1 × 10 ppm	–8.533	0.001^1)^	Sig < 0.05	Yes
	10 × 5 ppm	3.366	0.101	Sig > 0.05	No

Note: ^1)^ Sig < 0.05 indicates that it is significantly different (*p* < 0.05).

**Table 5.** Results of the tukey honestly significant differences (HSD) test for different doses on the inhibition zone of *S. aureus*.

Species	Treatment	Mean difference	Sig.	Indicators	Significant
*S. aureus*	1000 ppm × metanol	24.666	0.001^1)^	Sig < 0.05	Yes
	1000 × 1 ppm	16.033	0.001^1)^	Sig < 0.05	Yes
	1000 × 5 ppm	8.700	0.005^1)^	Sig < 0.05	Yes
	1000 × 10 ppm	4.766	0.119	Sig > 0.05	No
	1 × 5 ppm	–7.566	0.010^1)^	Sig < 0.05	Yes
	1 × 10 ppm	–11.266	0.001^1)^	Sig < 0.05	Yes
	10 × 5 ppm	3.700	0.284	Sig > 0.05	No

Note: ^1)^ Sig < 0.05 indicates that it is significantly different (*p* < 0.05).

**Table 6.** Results of the tukey honestly significant differences (HSD) test for different doses on the inhibition zone of *S. epidermidis*.

Species	Treatment	Mean difference	Sig.	Indicators	Significant
*S. epidermidis*	1000 ppm × metanol	24.666	0.001^1)^	Sig < 0.05	Yes
	1000 × 1 ppm	16.033	0.001^1)^	Sig < 0.05	Yes
	1000 × 5 ppm	8.133	0.039^1)^	Sig < 0.05	Yes
	1000 × 10 ppm	3.833	0.516	Sig > 0.05	No
	1 × 5 ppm	–5.200	0.254	Sig < 0.05	No
	1 × 10 ppm	–9.500	0.016^1)^	Sig < 0.05	Yes
	10 × 5 ppm	4.300	0.413	Sig > 0.05	No

Note: ^1)^ Sig < 0.05 indicates that it is significantly different (*p* < 0.05).

## Discussion

**Figure 4.**
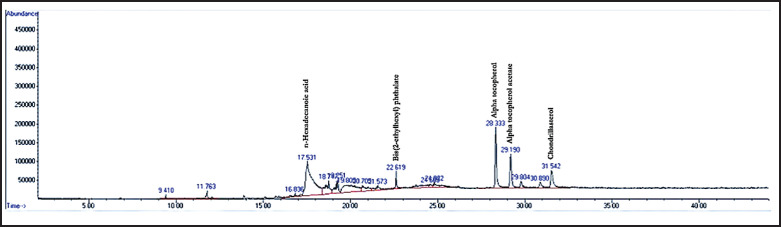
GC–MS chromatogram of *H. pluvialis* extract. Major peaks have been labeled with the names of corresponding compounds identified through NCBI. See also Table 1 for the full compound list and retention times.

Based on the study’s results, the first step in testing the content of bioactive compounds and anti-acne properties is to extract *H. pluvialis* to produce red oil extracts. Initially, maceration is the first step. The initial weight of *H. pluvialis* cell biomass before being soaked in acetone is 10 gm. Acetone was used as the extraction solvent due to its effectiveness in penetrating the thick sporopollenin-rich cell walls of *H*.* pluvialis* and its compatibility with carotenoid-rich compound recovery. Previous studies have shown that acetone yields higher astaxanthin recovery compared to ethanol or methanol [12,13]. The maceration solution was then filtered and evaporated using a rotary vacuum evaporator, yielding a total of 3.3 gm of *H. pluvialis* extract (Table 7). One of the quality parameters of the extract is the yield of the resulting extract. The yield is the ratio between the extract obtained and the initial sample. The yield is expressed in units of percent. In this study, the yield of *H. pluvialis* extract was 33%. According to Esati et al. [14] and Ghany et al. [15], the yield is considered good when it exceeds 10%. Therefore, the *H. pluvialis* extract obtained in this study was declared good because the yield was >10% [16]. After the extracts, the next step is to analyze the content of bioactive compounds using GC–MS. Seventeen bioactive compounds were identified in the peaks of *H. pluvialis* extracts using GC/MS analysis. Table 1 provides the results of the extraction of the bioactive compounds in tabular form. The chromatogram of the compounds identified by GC/MS is displayed in Figure 4. The mass spectra of the compounds were analyzed and identified using the NCBI database.

There were several chemical compounds in *H. pluvialis,* such as fatty acids, polysaccharides, vitamins, aromatic compounds, diterpenoids, DEHP, and other bioactive compounds. Fatty acids and another class of vitamin compounds, like alpha-tocopherol, were abundant in these algae. Across both fractions, fatty acids and vitamin compounds accounted for approximately 72.20% of the total peak percentage in *H. pluvialis*. Volatile compounds play a crucial role in giving algae their structure and flavor. Therefore, it is not surprising that researchers have studied the volatile compounds present in algae.

n-hexadecanoic acid has antibacterial and antifungal characteristics [17]. This fatty acid can affect immune responses through direct interaction with T cells. n-Hexadecanoic acid also provides anti-inflammatory effects by reducing the production of inflammatory mediators such as prostaglandin *E2*, *IL*-6, *IL*-*1β*, *TNF-*α, and nitric oxide [18]. This compound also plays a role in inhibiting the growth of acne. It has antioxidant, anti-inflammatory, and peptide properties. These compounds may be beneficial in treating acne, so *H. pluvialis* holds enormous potential for overcoming skin problems, especially acne [19].

**Table 7.** Yield results from *H. pluvialis* extract.

Solvent	Initial dry weight (gm)	Extract weight (gm)	Yield (%)
Acetone 100%	10	3.3	33

Advanced research conducted by Aparna et al. [20] identified the activity of n-Hexadecanoic acid as an anti-inflammatory agent, which was proven through significant inhibition in the kinetics study of the *PLA2* enzyme. This activity was attributed to the strong entropy-driven binding to the enzyme seen through ITC analysis. The formation of *PLA2* binary complex crystals in a 1:1 molar solution and its binding to the active side of the enzyme, as seen in the X-ray structure, demonstrates high binding affinity to the enzymatic active site. n-Hexadecanoic acid has various benefits, such as being an antioxidant, hypocholesterolemic, nematicide, pesticide, antiandrogenic, flavor, hemolytic, and 5-alpha reductase inhibitor [21]. In addition, Karunia et al. [22] found that n-Hexadecanoic acid can inhibit the growth of *S. aureus* bacteria.

Besides n-Hexadecanoic acid, the second most bioactive compound in *H. pluvialis* is alpha-tocopherol. Alpha-tocopherol is the main antioxidant in human skin, which can protect the skin from free radical damage [23]. In addition, this compound is also known to have anti-inflammatory properties that are beneficial in reducing inflammation in the skin. Alpha-tocopherol may help heal by accelerating skin regeneration [24]. Alpha-tocopherol has anti-inflammatory and antioxidant properties. It is very effective in treating acne-prone skin conditions. This compound helps with inflammation, one of the principal causes of acne. Thus, it may play a role in acne treatment and general skin care. This study focused on Gram-positive bacterial strains associated with acne, namely *P. acnes*, *S. aureus*, and *S*. *epidermidis*. As no Gram-negative bacteria were included in the experimental design, the specificity of the extract against different bacterial classes cannot be conclusively determined. The current study conveys the findings of investigations regarding the content of bioactive compounds with the potential to inhibit acne-causing bacteria using the disc-diffusion assay on *P*. *acnes*, *S. aureus*, and *S*. *epidermidis*. *H. pluvialis *extract can create inhibition zones, indicating its bioactive compounds’ ability to suppress the growth of pathogenic bacteria.

Microalgae *H. pluvialis* is a promising source of bioactive compounds and has become a key focus in the creation of various medicinal drugs. In a study, algae extracts prepared with organic solvents were tested against three microorganisms, showing antibacterial properties (Table 2). The results revealed that microalgae extracts contained numerous antibacterial compounds. Our results indicate that the *H. pluvialis* extract demonstrated greater antimicrobial activity than a 1,000 ppm chloramphenicol extract.

Chloramphenicol was selected as the positive control due to its broad-spectrum antibacterial activity and its common use in laboratory-based antimicrobial testing [25]. The results of this study show that *H. pluvialis* extracts exhibited the highest level and broadest spectrum of antibacterial activity against the tested microorganisms. The disc diffusion assay results by paper disc exhibited a similar trend, with antibacterial efficiency increasing as the concentration increased.

These variations may be due to the different solubility behaviors of the bioactive compounds. Several factors may have influenced the antibacterial potency of microalgae, including the efficiency of extraction, the type of solvent used, and the resistance of the bacteria tested, all of which could have contributed to the observed variations in the results [26]. The potential mechanisms by which the paper discs inhibit *P*. *acnes*, *S. aureus*, and *S*. *epidermidis* may be attributed to the primary compounds extracted from *H*. *pluvialis* (fatty acids, vitamins, polysaccharides, aromatic compounds, and diterpenoids). These compounds, present in the paper discs, penetrate the bacterial cell walls, leading to dysfunction in the bacterial cells and, ultimately, cell death. Although this study did not directly observe membrane damage through imaging techniques such as SEM, the proposed mechanism of membrane penetration is supported by previous studies showing that carotenoid- and lipid-rich extracts can compromise bacterial cell wall integrity through increased permeability and oxidative damage [27].

The antibacterial properties and structure of bacteria and algae depend on the processes used to utilize freshwater algae, both macroalgae and microalgae, as antimicrobial agents [28] and [29]. The weak antibacterial activity observed at 1 ppm may be due to the insufficient concentration of active constituents to reach the minimum inhibitory threshold. At such low concentrations, the bioactive compounds may exhibit reduced solubility or increased molecular aggregation, limiting their diffusion in the agar medium and reducing contact with bacterial cells. Similar concentration-dependent effects have been reported in previous studies using natural extracts [30]. Antibiotics are often less effective against Gram-negative bacteria due to the strong, rigid structure of their cell walls, making them more complex than Gram-positive bacteria. As a result, it is more difficult for active compounds such as β-lactams, quinolones, and other antibiotics to penetrate the bacterial cells and exert their antibacterial effects [28]. The results showed that Gram-positive bacteria were significantly more resistant to antibacterial activity than Gram-negative bacteria. This resistance is due to the structure and components of their cell walls, as well as the presence of short-chain fatty acids [29].

Although the exact mechanism by which fatty acids prevent the entry of active compounds from algae extracts is still unclear, numerous studies have found that fatty acids and lipids contribute to the breakdown of cellular membranes [31–33]. The study concluded by emphasizing the potential of naturally occurring microalgae as a possible antibiotic against various harmful and disease-causing bacteria [34,35]. Although some of the identified compounds (n-hexadecanoic acid and alpha tocopherol) have been previously reported to possess anti-inflammatory properties, the current study did not include direct assays (e.g., ROS scavenging, cytokine analysis, or biofilm disruption) to confirm such activity. Further studies are warranted to investigate the mechanistic pathways involved.

## Conclusion

This study describes the existence of antibacterial chemicals in microalgae. Algae exhibit varying antibacterial properties depending on their class. In terms of the extraction method, ethanol was the solvent that, in some cases, enhanced the activity of the extracts against the bacterial strain used in the antibiotic test. *Haematococcus pluvialis* was extracted and used to synthesize antibacterial materials for anti-acne. Results of the paper disc confirmed that it had potential inhibitions on *P*. *acnes*, *S. aureus,* and *S*. *epidermidis*. The carotenoid-rich extract of *H*. *pluvialis* demonstrated significant antibacterial activity against Gram-positive acne-related bacteria. However, since Gram-negative bacteria were not tested in this study, the antimicrobial spectrum of the extract cannot be generalized beyond the tested strains. The findings suggest that microalgae from *H*. *pluvialis* hold potential as sources of bioactive compounds and should be further investigated for the isolation of natural antibiotics. *Haematococcus pluvialis* shows promising potential for future pharmaceutical and acne treatment applications.

## List of abbreviations

ATCC, The American Type Culture Collection; DEHP, Di-(2-ethylhexyl) phthalate; GC–MS, Gas chromatography–mass spectrometry; NCBI, National Center for Biotechnology Information; pH, potential of Hydrogen; ROS, Reactive Oxygen Species
